# Heart rate variability analysis for the prediction of EEG grade in infants with hypoxic ischaemic encephalopathy within the first 12 h of birth

**DOI:** 10.3389/fped.2022.1016211

**Published:** 2023-01-04

**Authors:** Andreea M Pavel, Sean R Mathieson, Vicki Livingstone, John M O’Toole, Ronit M Pressler, Linda S de Vries, Janet M Rennie, Subhabrata Mitra, Eugene M Dempsey, Deirdre M Murray, William P Marnane, Geraldine B Boylan, ANSeR Consortium

**Affiliations:** ^1^ INFANT Research Centre, University College Cork, Cork, Ireland; ^2^ Department of Paediatrics and Child Health, University College Cork, Cork, Ireland; ^3^ Department of Clinical Neurophysiology, Great Ormond Street Hospital for Children NHS Trust, London, United Kingdom; ^4^ Utrecht Brain Center, University Medical Center Utrecht, Utrecht University, Utrecht, Netherlands; ^5^ Institute for Women's Health, University College London, London, United Kingdom; ^6^ School of Engineering, University College Cork, Cork, Ireland

**Keywords:** newborn, neonatal encephalopathy, heart rate variability, electroencephalography, electrocardiogram

## Abstract

**Background and aims:**

Heart rate variability (HRV) has previously been assessed as a biomarker for brain injury and prognosis in neonates. The aim of this cohort study was to use HRV to predict the electroencephalography (EEG) grade in neonatal hypoxic-ischaemic encephalopathy (HIE) within the first 12 h.

**Methods:**

We included 120 infants with HIE recruited as part of two European multi-centre studies, with electrocardiography (ECG) and EEG monitoring performed before 12 h of age. HRV features and EEG background were assessed using the earliest 1 h epoch of ECG-EEG monitoring. HRV was expressed in time, frequency and complexity features. EEG background was graded from 0-normal, 1-mild, 2-moderate, 3-major abnormalities to 4-inactive. Clinical parameters known within 6 h of birth were collected (intrapartum complications, foetal distress, gestational age, mode of delivery, gender, birth weight, Apgar at 1 and 5, assisted ventilation at 10 min). Using logistic regression analysis, prediction models for EEG severity were developed for HRV features and clinical parameters, separately and combined. Multivariable model analysis included 101 infants without missing data.

**Results:**

Of 120 infants included, 54 (45%) had normal-mild and 66 (55%) had moderate-severe EEG grade. The performance of HRV model was AUROC 0.837 (95% CI: 0.759–0.914) and clinical model was AUROC 0.836 (95% CI: 0.759–0.914). The HRV and clinical model combined had an AUROC of 0.895 (95% CI: 0.832–0.958). Therapeutic hypothermia and anti-seizure medication did not affect the model performance.

**Conclusions:**

Early HRV and clinical information accurately predicted EEG grade in HIE within the first 12 h of birth. This might be beneficial when EEG monitoring is not available in the early postnatal period and for referral centres who may want some objective information on HIE severity.

## Introduction

Despite developments in feto-maternal monitoring and care, hypoxia-ischaemia (HIE) remains the main cause of neonatal encephalopathy with an incidence of up to 1.5–3 per 1000 live births in high-income countries and significantly higher in low-middle income countries (1, 2). Therapeutic hypothermia is the only available treatment which has been shown to improve outcomes for moderate and severe HIE (3–6). To optimise the beneficial effects of hypothermia, treatment has to start as soon as possible after birth (7).

Sometimes, the early clinical picture alone is insufficient to decide if a newborn will benefit from therapeutic hypothermia and many neonatal centres use electroencephalography (EEG) to help assess the severity of encephalopathy. The use of EEG monitoring in neonatal units for diagnosis, treatment monitoring and prognosis is increasing worldwide (8, 9). International guidelines recognise the benefit of EEG for improved neonatal outcomes and recommend EEG for accurate diagnosis and monitoring of high risk infants (10, 11). However, introducing neonatal EEG monitoring is challenging; equipment is expensive and specialised personnel are required 24/7 to interpret the EEG. This is not readily available for most neonatal units, especially in low-income countries, thus the need of other methods to help identify those most at risk (12, 13).

Due to the widespread use of electrocardiography (ECG) monitoring, heart rate variability (HRV) has been assessed as a possible biomarker for brain injury and prognosis in neonatal populations (14–17). The naturally occurring variation in heartbeat can be described by HRV analysis. The autonomic nervous system, through sympathetic and parasympathetic control regulates HRV. Changes in beat-to-beat heart rate have been correlated with stress levels. A decrease in HRV was associated with stress elevation and studies suggest that HRV could predict HIE severity and outcome (18–21). In this study we investigated if HRV can predict EEG grade in infants with HIE in the first 12 h after birth. We hypothesised that HRV analysis may be a useful, objective tool to indicate EEG encephalopathy grade in HIE, which could be beneficial when EEG is not yet available. The aim of this study was to assess the use of early HRV analysis for the prediction of EEG encephalopathy grade in newborn infants.

## Methods

### Study setting and participants

The current study presents the results of a secondary analysis of data from two multi-centre cohort studies (ClinicalTrials.gov Identifier: NCT02160171 and NCT02431780) (22, 23) which recruited newborn infants requiring EEG monitoring across eight European tertiary Neonatal Units. Both studies had ethical approval granted by national and local ethics committees and parental/legal guardian written consent was obtained.

Newborn infants with the following were included: 36 weeks gestation or greater with presumed HIE and EEG-ECG monitoring for at least one hour before 12 h of age. The diagnosis of HIE was initially established based on signs of perinatal asphyxia and encephalopathy on neurological examination (modified Sarnat score within 24 h of age) and retrospectively corroborated with abnormalities suggestive of HIE on EEG and brain MRI. The clinical grade of HIE was based on the most severe score of modified Sarnat score. Infants with sepsis, meningitis, stroke, metabolic or genetic encephalopathy were excluded. Infants were also excluded if seizures were detected before, during or at least one hour after the EEG-ECG epoch selected for analysis.

### EEG and ECG monitoring

EEG monitoring commenced as soon as possible after birth, using disposable electrodes according to the 10:20 electrode placement system for neonates (F3, F4, C3, C4, Cz, T3, T4, O1/P3 and O2/P4). EEG recording sampling rate was 250 Hz or 256 Hz and filter bandwidth was 0.5 to 70 Hz. Separate electrodes on each shoulder of the newborn were used for single channel ECG monitoring synchronised with EEG. The EEG machines used were: NicoletOne ICU Monitor (Natus, United States), Nihon Kohden EEG (Neurofax EEG-1200, Japan) or XLTek EEG (Natus, United States).

The earliest one-hour epoch of EEG-ECG was extracted for each infant, within 12 h of age.

### EEG analysis and grading of HIE

EEG background was graded using a system described previously by our group: 0-normal EEG background, 1-mild abnormalities, 2-moderate abnormalities, 3-major abnormalities and 4-inactive EEG background (24).

### HRV analysis

In-house software (HRV Analysis, Beta Version 1.12, ©University College Cork 2008–2012) was used to automatically identify R-peaks on ECG. A manual correction was applied after visual inspection to ensure all R-peaks were correctly identified and artefacts were annotated and removed from analysis. The RR interval was generated as the time difference between each R peak. HRV features were calculated for five-minute segments with 50% overlap, using the same procedure described in previous studies (19, 25–27). If more than 50% of a five-minute segment was artefact, this was removed. Across all 1 h epochs included in the analysis a median (IQR) of 1.2% (0.2% to 4.4%) was removed from the analysis. Features were summarised by median value over all segments. HRV was expressed in time, frequency and complexity features. The time domain features were mean NN-interval (the normalised RR interval), standard deviation of the NN-interval (SDNN), triangular interpolation of NN-interval histogram (TINN). The frequency domain features were high frequency power (HF, 0.2 to 2 Hz), low frequency power (LF, 0.04 to 0.2 Hz), very low frequency power (VLF, 0.01 to 0.04 Hz) and the low frequency/high frequency (LF/HF) ratio. The complexity features were calculated from multiscale entropy. The moving-average filter was used to generate the HRV at different scales, then entropy was calculated for each of these multiscale HRV signals (25, 26). Entropy was estimated using sample entropy with an embedding dimension of 2 and a tolerance of 0.15 (25). Four features were calculated from the plot of scale vs. entropy: 1) complexity index, estimated as the total area under the multiscale entropy curve, 2) maximum entropy, 3) slope of multiscale entropy over short-duration scale factors, by fitting a line to the entropy curve from scales 1 to 5 then calculating the slope of this line, and 4) slope of long-duration scale factors, the slope of a line fitted to the 6 to 20 scale factors (25, 26).

### Clinical parameters

Several clinical features known within 6 h of birth were collected: presence of intrapartum complications, suspected foetal distress predelivery, gestational age, delivery mode, gender, birth weight, Apgar scores (1 and 5 min), need for assisted ventilation at 10 min of age, cord pH, first postnatal lactate, first postnatal base deficit. We considered intrapartum complications and their consequences as per the following: placental abruption, ruptured uterus, vasa praevia, intrapartum haemorrhage, cord accident or prolapse, shoulder dystocia, meconium-stained liquor or other (poor progression, HELLP syndrome/Eclampsia, breech presentation, reduced foetal movements, foetal bradycardia, prolonged rupture of membranes). These clinical parameters were used to predict the EEG grade of encephalopathy. Mode of delivery was combined into emergency (assisted vaginal delivery and emergency caesarean section) and non-emergency (unassisted vaginal delivery and elective caesarean section). The clinical management of each infant (including the need for therapeutic hypothermia and anti-seizure medication) was decided by the local clinical teams at each study site.

### Statistical analysis

Categorical variables were described using frequencies and percentages and continuous variables using means and standard deviations (SDs), when data was normally distributed or medians and inter-quartile ranges (IQRs) otherwise. We investigated the ability of HRV features and clinical parameters (alone and in combination) to predict EEG encephalopathy grade using univariable and multivariable logistic regression analysis. The EEG grade was classified as a dichotomous variable, normal-mild group (grades 0 to 1, outcome = 0) and moderate-severe group (grades 2 to 4, outcome = 1). Univariable logistic regression analysis investigated the prediction of each variable for outcome (moderate-severe EEG grade). Using multivariable logistic regression analysis, we combined the variables with the best prediction for outcome and developed a HRV prediction model, a clinical prediction model and a combined model (HRV and clinical model). Variables with *p* < 0.25 in univariable analyses were eligible for inclusion in multivariable models. If variables were highly correlated, the variable with the highest area under receiver operating characteristic curve (AUROC) in the univariable analysis was included in the multivariable model. The Hosmer-Lemeshow goodness of fit test was used to determine the fit of the multivariable models with *p* < 0.05 indicating lack of fit. The following clinical parameters had missing data: suspected foetal distress in labour (14 (11.7%) infants), Apgar score at 1 min (4 (3.3%) infants), 5 min (4 (3.3%) infants) and 10 min 15 (12.5% infants), pH (18 (15%) infants), lactate (35 (29.2%) infants), base deficit (31 (25.8%) infants). For accurate comparison between models, the multivariable analysis included 101 infants with no missing clinical data. Subgroup analyses were performed exclusively with infants who received therapeutic hypothermia (*n* = 97 infants) and with infants not on anti-seizure medication prior to selected epoch (*n* = 71 infants). Due to missing data, cord pH, lactate and base deficit could not be included in the main multivariable analysis. However, the subgroup analyses also included the information on blood gases. Prior to performing logistic regression analyses, positively skewed HRV features were first log transformed and then all features were standardised to make the regression coefficients comparable. The AUROC and its corresponding 95% confidence interval (CI) were calculated from the univariable and multivariable models to assess their ability to predict EEG grade. An AUROC can range from 0.5 (discrimination no better than chance) to 1 (perfect discrimination). For multivariable models, Youden's index (index = sensitivity + specificity-1) was used to find the optimal sensitivity-specificity cut-off point on the AUROC curve and the corresponding sensitivity, specificity, positive predictive value (PPV) and negative predictive value (NPV) were estimated.

All tests were two-sided and a *p* < 0.05 was considered statistically significant. IBM SPSS Statistics (version 25.0, IBM Corp., Armonk, NY, United States) was used for the statistical analysis AUC curves were drawn using Stata (version 17.0, StataCorp, LP College Station, TX, USA).

## Results

Out of the two original cohorts, 120 infants with HIE had at least one hour of EEG-ECG monitoring and were included in this analysis: 54 (45%) had a normal-mild EEG grade and 66 (55%) had a moderate-severe EEG grade on earliest EEG epoch. The two groups were similar in terms of gestational age, birth weight, gender, age at start of EEG monitoring and age when EEG epoch was analysed. Compared with the normal-mild EEG group, the moderate-severe EEG group had lower Apgar scores at 1 min (median (IQR) 2 (1 to 4) vs. 1 (0 to 2)), at 5 min (median (IQR) 5 (4 to 7) vs. 3 (1 to 4)) and at 10 min (median (IQR) 7 (5 to 9) vs. 4 (3 to 5)), had lower pH (mean (SD) 7.05 (0.15) vs. 6.95 (0.20)), higher lactate (mean (SD) 10.9 (3.2) vs. 13.0 (5.2)), higher base deficit (mean (SD) 14.6 (4.5) vs. 17.0 (6.4)), higher need for ventilation (22 (41.5%) vs. 58 (89.2%)), more infants were cooled (33 (61.1%) vs. 64 (97%)), and more infants had electrographic seizures (6 (11.1%) vs. 34 (51.5%)). Demographic data is presented in Table 1.

**Table 1:** Study cohort demographics.

	*n*	All infants	Normal-mild EEG background group	Moderate-severe EEG background group
		*n* = 120^a^	*n* = 54^a^	*n* = 66^a^
Gestational age at birth (weeks), median (IQR)		40.1 (39.2 to 41.0)	40.6 (39.4 to 41.1)	40.0 (39.0 to 40.9)
Mode of delivery, *n* (%)				
Unassisted vaginal delivery		39 (32.5)	13 (24.1)	26 (39.4)
Assisted vaginal delivery		43 (35.8)	25 (46.3)	18 (27.3)
Elective caesarean section		6 (5.0)	1 (1.9)	5 (7.6)
Emergency caesarean section		32 (26.7)	15 (27.8)	17 (25.8)
Birth weight (g), mean (SD)		3,475 (607)	3,525 (492)	3,434 (689)
Gender, male *n* (%)		75 (62.5)	32 (59.3)	43 (65.2)
Apgar score at 1 min, median (IQR)	116	1 (0 to 3)	2 (1 to 4)	1 (0 to 2)
Apgar score at 5 min, median (IQR)	116	4 (2 to 6)	5 (4 to 7)	3 (1 to 4)
Apgar score at 10 min, median (IQR)	105	5 (4 to 8)	7 (5 to 9)	4 (3 to 5)
Adrenaline during resuscitation, yes *n* (%)		20 (16.6)	3 (5.6)	17 (25.8)
Assisted ventilation at 10 min of age, yes *n* (%)	118	80 (67.8)	22 (41.5)	58 (89.2)
Lowest cord pH, mean (SD)	102	7.0 (0.2)	7.1 (0.2)	7.0 (0.2)
First postnatal lactate, mean (SD)	85	11.90 (4.35)	10.9 (3.2)	13 (5.2)
First postnatal base deficit, mean (SD)	89	15.8 (5.6)	14.6 (4.5)	17 (6.4)
HIE clinical grade at discharge				
Mild, *n* (%)		51 (42.5)	40 (74.1)	11 (16.7)
Moderate, *n* (%)		44 (36.7)	13 (24.1)	31 (47.0)
Severe, *n* (%)		25 (20.8)	1 (1.9)	24 (36.4)
Therapeutic hypothermia (yes), *n* (%)		97 (80.8)	33 (61.1)	64 (97.0)
Age at start of therapeutic hypothermia (hours), median (IQR)	97	1 (1 to 3)	2 (1 to 3)	1 (1 to 4)
Age at start of EEG monitoring (hours), median (IQR)		4.4 (3.2 to 7.4)	4.3 (3.2 to 7.4)	4.7 (3.3 to 7.7)
Age at start of EEG epoch (hours), median (IQR)		5.0 (4.1 to 8.3)	5 (4.1 to 8.2)	6.5 (4.2 to 8.6)
Electrographic seizures (yes), *n* (%)		40 (33.3)	6 (11.1)	34 (51.5)
Any anti-seizure medication given before EEG epoch analysed (yes), *n* (%)		19 (15.8)	0	19 (28.8)

EEG, electroencephalography.

^a^ unless otherwise stated.

### Univariable analysis

Out of the HRV features, a higher mean NN and lower LF/HF ratio, TINN, MSE complexity index, MSE short-scale slope and maximum MSE were associated with moderate-severe EEG grade (analysis presented in Table 2).

**Table 2:** HRV univariable analysis.

HRV Features	Normal-mild EEG group (median, IQR)	Moderate-severe EEG group (median, IQR)	OR^a^ (95% CI)	*p* value	AUROC (95% CI)
	*n* = 54	*n* = 66			
Mean NN (ms)^b^	523.3 (481.9 to 603.6)	591.6 (532.6 to 692.6)	1.97 (1.30–2.97)	0.001	0.675 (0.579–0.772)
SDNN (ms)^c^	18.0 (13.6 to 34.0)	19.8 (10.6 to 33.8)	0.93 (0.65–1.34)	0.712	0.504 (0.400–0.609)
VLF power (ms^2^)^c^	1772.5 (678.3 to 4452.1)	1835.8 (495.9 to 4308.9)	0.79 (0.54–1.15)	0.211	0.544 (0.441–0.648)
LF power (ms^2^)^c^	271.0 (65.3 to 719.9)	194.6 (47.4 to 958.3)	0.89 (0.62–1.28)	0.529	0.526 (0.422–0.629)
HF power (ms^2^)^c^	3.8 (1.1 to 18.6)	8.0 (2.3 to 43.2)	1.38 (0.95–2.00)	0.091	0.585 (0.483–0.688)
LF/HF ratio^c^	48.9 (24.6 to 73.6)	18.4 (7.8 to 37.1)	0.40 (0.25–0.64)	<0.001	0.721 (0.629–0.813)
TINN (ms)^c^	62.5 (46.9 to 95.7)	56.6 (31.3 to 78.1)	0.66 (0.45–0.97)	0.035	0.597 (0.495–0.698)
MSE Complexity Index^b^	26.5 (23.4 to 29.6)	23.5 (18.9 to 25.8)	0.46 (0.29–0.73)	0.001	0.695 (0.600–0.790)
MSE short-scale slope^b^	0.11 (0.05 to 0.14)	0.04 (-0.01 to 0.07)	0.26 (0.15–0.47)	<0.001	0.776 (0.692–0.860)
MSE long-scale slope^b^	-0.01 (-0.03 to 0.03)	0.00 (-0.02 to 0.03)	1.14 (0.79–1.64)	0.477	0.555 (0.447–0.663)
MSE maximum^b^	2.4 (2.2 to 2.6)	2.2 (1.9 to 2.5)	0.39 (0.22–0.69)	0.001	0.690 (0.596–0.783)

^a^ For the non-transformed variables the odds ratio represents the change in odds for a one-standard deviation increase in the HRV variable. For the transformed variables the odds ratio represents the change in odds for a one-standard deviation increase in the log HRV variable.

^b^ HRV variable was standardised prior to conducting the logistic regression.

^c^ HRV variable was log transformed (log base 10) and then standardised prior to conducting the logistic regression.

HRV, Heart Rate Variability; EEG, electroencephalography; NN interval, normalised RR interval; SDNN, standard deviation of the NN interval; VLF power, very low frequency power (≤ 0.04 Hz); LF power, low frequency power (0.04–0.15 Hz); HF power, high frequency power (0.15–0.4 Hz), LF/HF ratio, low frequency/high frequency ratio; TINN, triangular interpolation of the NN interval histogram; MSE, multiscale entropy.

Out of the clinical parameters, non-emergency delivery mode, lower Apgar scores, higher need for ventilation at 10 min, lower pH and higher lactate and base deficit were associated with moderate-severe EEG grade (analysis presented in Table 3).

**Table 3:** Clinical univariable analysis.

Clinical Parameters	Normal-mild EEG group	Moderate-severe EEG group	OR (95% CI)	*p* value	AUROC (95% CI)
	*n* = 54^a^	*n* = 66^a^			
Any Intrapartum complications, yes *n* (%)	46 (85.2)	57 (86.4)	1.10 (0.39–3.08)	0.854	0.506 (0.402–0.610)
Suspected foetal distress in labour, yes *n* (%)^b^	40 (81.6)	39 (68.4)	0.49 (0.20–1.22)	0.123	0.566 (0.457–0.675)
Gestational age at birth (weeks), median (IQR)	40.6 (39.4 to 41.1)	40.0 (39.0 to 40.9)	0.78 (0.59–1.05)	0.099	0.591 (0.489–0.694)
Mode of delivery, emergency *n* (%)	40 (74.1)	35 (53.0)	0.40 (0.18–0.86)	0.019	0.605 (0.504–0.707)
Gender, male *n* (%)	32 (59.3)	43 (65.2)	1.29 (0.61–2.70)	0.507	0.529 (0.425–0.634)
Birth weight (g), mean (SD)	3,525 (492)	3,434 (689)	0.98 (0.92–1.04)^h^	0.410	0.570 (0.468–0.673)
Apgar scores at 1 min, median (IQR)^c^	2 (1 to 4)	1 (0 to 2)	0.71 (0.57–0.88)	0.002	0.708 (0.614–0.802)
Apgar scores at 5 min, median (IQR)^c^	5 (4 to 7)	3 (1 to 4)	0.67 (0.55–0.81)	<0.001	0.756 (0.667–0.846)
Assisted ventilation at 10 min of age, yes *n* (%)^d^	22 (41.5)	58 (89.2)	11.68 (4.49–30.36)	<0.001	0.739 (0.644–0.833)
Lowest cord pH, mean (SD)^e^	7.05 (0.15)	6.95 (0.20)	0.97 (0.95–0.99)^i^	0.008	0.666 (0.562–0.770)
First postnatal lactate, mean (SD)^f^	10.9 (3.2)	13.0 (5.2)	1.13 (1.01–1.25)	0.029	0.644 (0.519–0.768)
First postnatal base deficit mean (SD)^h^	14.6 (4.5)	17.0 (6.4)	1.08 (1.00–1.17)	0.050	0.658 (0.539–0.777)

^a^ unless otherwise stated.

^b^ *n* = 49 in normal-mild group and *n* = 57 in moderate-severe group.

^c^ *n* = 52 in normal-mild group and *n* = 64 in moderate-severe group.

^d^ *n* = 53 in normal-mild group and *n* = 65 in moderate-severe group.

^e^ *n* = 45 in normal-mild group and *n* = 57 in moderate-severe group.

^f^ *n* = 44 in normal-mild group and *n* = 41 in moderate-severe group.

^g^ *n* = 46 in normal-mild group and *n* = 43 in moderate-severe group.

^h^ The odds ratio represents the change in odds for a 100 g increase in birthweight.

^i^ The odds ratio represents the change in odds for a 0.01 increase in cord pH.

Mode of delivery was defined as emergency (assisted vaginal delivery and emergency caesarean section) and non-emergency (unassisted vaginal delivery and elective caesarean section).

### Multivariable analysis

**Figure 1:**
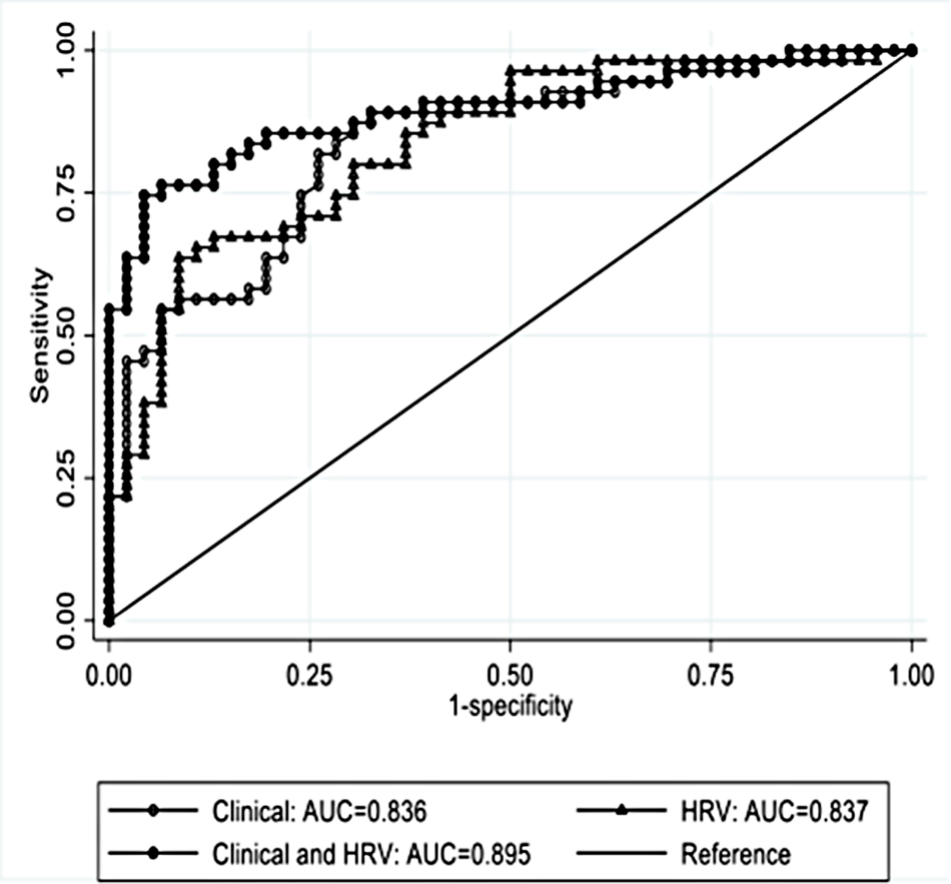
The AUROC of the multivariable analysis for HRV and clinical prediction model.

For comparability of the multivariable models, we report the results of the multivariable analysis restricted to 101 infants with complete clinical information, which were included in the clinical prediction model. The AUROC for the multivariable HRV prediction model was 0.837 (95% CI:0.759–0.914, *p* < 0.001), Table 4. Based on Youden's index, the optimal cut-off was *p* = 0.66, giving sensitivity 63.6%, specificity 91.3%, PPV 89.7% and NPV 67.7%. The AUROC for the multivariable clinical prediction model was 0.836 (95% CI:0.759–0.914, *p* < 0.001), with sensitivity 87.3%, specificity 69.6%, PPV 77.4% and NPV 82.1% using the optimal cut-off *p* = 0.57, Table 5. The combined HRV and clinical model, had the best performance with an AUROC of 0.895 (95% CI:0.832–0.958, *p* < 0.001) (Table 6), with sensitivity 74.6%, specificity 95.7%, PPV 95.3% and NPV 75.9% (optimal cut-off *p* = 0.66). Performance of prediction models is shown in Figure 1.

**Table 4:** HRV multivariable analysis (HRV prediction model), *n* = 101 infants.

	OR^a^ (95% CI)	*p* value	AUROC (95% CI)
Mean NN (ms)^b^	2.73 (1.34–5.53)	0.005	0.837 (0.759–0.914)
HF power (ms^2^)^c^	1.45 (0.44–4.81)	0.544	
LF/HF ratio^c^	1.48 (0.51–4.34)	0.471	
TINN (ms)^c^	0.61 (0.19–1.93)	0.398	
MSE Complexity Index^b^	0.60 (0.32–1.13)	0.115	
MSE slope short^b^	0.31 (0.12–0.79)	0.014	

^a^ For the non-transformed variables the odds ratio represents the change in odds for a one-standard deviation increase in the HRV variable. For the transformed variables the odds ratio represents the change in odds for a one-standard deviation increase in the log HRV variable.

^b^ HRV variable was standardised prior to conducting the logistic regression.

^c^ HRV variable was log transformed (log base 10) and then standardised prior to conducting the logistic regression.

The multivariable model is: log (*p*/(1 − *p*)) = 0.49 + 1.00 (standardized Mean NN) + 0.37 (standardised log 10 HF power) + 0.40 (standardised log 10 LF HF ratio) −0.50 (standardised log 10 TINN) − 0.51 (standardised MSE c index) −1.16 (standardised MSE slope short) *p* = 0.480 for Hosmer-Lemeshow goodness of fit.

HRV, Heart Rate Variability; NN interval, normalised RR interval; HF power, high frequency power (0.15–0.4 Hz), LF/HF ratio, low frequency/high frequency ratio; TINN, triangular interpolation of the NN interval histogram; MSE, multiscale entropy.

**Table 5:** Clinical multivariable analysis (clinical prediction model), *n* = 101 infants.

	OR (95% CI)	*p* value	AUROC (95% CI)
		
Suspected foetal distress in labour (yes)	0.28 (0.06–1.39)	0.120	0.836 (0.759–0.914)
Gestational age (weeks)	0.78 (0.52–1.17)	0.233	
Mode of delivery (emergency)	0.73 (0.20–2.58)	0.619	
Apgar scores at 5 min	0.93 (0.74–1.17)	0.532	
Assisted ventilation at 10 min of age (yes)	14.92 (3.81–58.35)	<0.001	

The multivariable model is: log[*p*/(1-*p*)] = 9.71-1.26 (suspected foetal distress in labour)-0.25 (GA at delivery) −0.32 (emergency delivery) – 0.07 (Apgar score at 5 min) + 2.70 (assisted ventilation) *p* = 0.325 for Hosmer-Lemeshow goodness of fit.

**Table 6:** HRV and clinical prediction model, *n* = 101 infants.

	OR (95% CI)	*p* value	AUROC (95% CI)
		
Mean NN (ms)^a^	2.34 (1.07–5.10)	0.032	0.895 (0.832–0.958)
HF power (ms^2^)^a^	1.94 (0.47–7.99)	0.361	
LF/HF ratio^b^	1.51 (0.47–4.89)	0.488	
TINN (ms)^b^	0.70 (0.19–2.58)	0.595	
MSE Complexity Index^a^	0.84 (0.38–1.84)	0.661	
MSE short-scale slope^a^	0.35 (0.13–0.98)	0.045	
Suspected foetal distress in labour (yes)	0.30 (0.04–2.20)	0.237	
Gestational age (weeks)	0.76 (0.48–1.22)	0.251	
Mode of delivery (emergency)	0.63 (0.12–3.29)	0.583	
Apgar scores at 5 min	0.94 (0.72–1.23)	0.654	
Assisted ventilation at 10 min of age (yes)	8.94 (1.86–43.00)	0.006	

^a^ HRV variable was standardised prior to conducting the logistic regression.

^b^ HRV variable was log transformed (log base 10) and then standardised prior to conducting the logistic regression.

The multivariable model is: log[*p*/(1-*p*)] = 11.49 + 0.85 (standardised Mean NN) + 0.66 (standardised log 10 HF power) + 0.42 (standardised log 10 LF/HF ratio) – 0.35 (standardised log 10 TINN)-0.18 (standardised MSE c index) −1.04 (standardised MSE slope short) −1.20 (suspected foetal distress in labour)-0.28 (GA at delivery) −0.46 (emergency delivery) – 0.06 (Apgar score at 5 min) + 2.19 (assisted ventilation) *p* = 0.548 for Hosmer-Lemeshow goodness of fit.

HRV, Heart Rate Variability; NN interval, normalised RR interval; HF power, high frequency power (0.15–0.4 Hz), LF/HF ratio, low frequency/high frequency ratio; TINN, triangular interpolation of the NN interval histogram; MSE, multiscale entropy.

To account for the confounding effect of therapeutic hypothermia and anti-seizure medication, we analysed data exclusively from infants undergoing therapeutic hypothermia and from infants which did not receive anti-seizure medication prior to the selected epoch. For these subgroup analyses we also included data on blood gases (pH, lactate and base deficit). Predictive performance of the models including only infants that received therapeutic hypothermia (*n* = 97 infants) was: HRV model AUROC 0.812 (95% CI:0.726–0.898), clinical model AUROC 0.781 (95% CI:0.658–0.905), HRV and clinical model AUROC 0.864 (95% CI:0.766–0.963) (Table 7). Models from the subgroup of infants without anti-seizure medication given prior to selected ECG-EEG epoch (*n* = 71 infants) were: HRV model AUROC 0.821 (95% CI:0.721–0.922), clinical model AUROC 0.829 (95% CI:0.736–0.923), HRV and clinical model AUROC 0.883 (95% CI:0.800–0.965) (Table 8).

**Table 7:** Subgroup analysis of infants undergoing therapeutic hypothermia - HRV and clinical prediction model, *n* = 52 infants.

	OR (95% CI)	*p* value	AUROC (95% CI)
		
Mean NN (ms)^a^	1.72 (0.50–5.86)	0.386	0.864 (0.766–0.963)
LF/HF rate^b^	1.70 (0.49–5.90)	0.401	
TINN (ms)^b^	1.16 (0.43–3.12)	0.769	
MSE short-scale slope^a^	0.27 (0.06–1.30)	0.102	
MSE maximum^a^	1.88 (0.44–8.01)	0.395	
Suspected foetal distress in labour (yes)	0.11 (0.00–2.77)	0.178	
Gestational age (weeks)	0.80 (0.38–1.69)	0.562	
Mode of delivery (emergency)	3.19 (0.17–58.73)	0.434	
Gender (male)	1.54 (0.29–8.06)	0.608	
Apgar scores at 5 min	0.79 (0.51–1.22)	0.289	
Assisted ventilation at 10 min of age (yes)	4.03 (0.28–58.46)	0.307	
Cord pH	0.01 (0.00–2.37)	0.103	
Base deficit	0.97 (0.79–1.19)	0.741	

^a^ HRV variable was standardised prior to conducting the logistic regression.

^b^ HRV variable was log transformed (log base 10) and then standardised prior to conducting the logistic regression.

HRV, Heart Rate Variability; NN interval, normalised RR interval; LF/HF ratio, low frequency/high frequency ratio; TINN, triangular interpolation of the NN interval histogram; MSE, multiscale entropy.

**Table 8:** Subgroup analysis of infants without anti-seizure medication prior to EEG epoch analysed - HRV and clinical prediction model, *n* = 56 infants.

	OR (95% CI)	*p*	AUROC (95% CI)
Mean NN (ms)^a^	2.23 (0.58–8.58)	0.242	0.892 (0.793-0.990)
HF power (ms2)^b^	1.75 (0.18–16.98)	0.629	
LF/HF ratio^b^	1.40 (0.18–10.89)	0.751	
TINN (ms)^b^	1.18 (0.19–7.42)	0.858	
MSE Complexity Index^a^	1.97 (0.41–9.56)	0.400	
MSE short-scale slope^a^	0.31 (0.05–1.89)	0.202	
MSE long-scale slope^a^	1.07 (0.26–4.41)	0.922	
Gestational age (weeks)	0.76 (0.34–1.69)	0.499	
Birth weight (g)^c^	1.09 (0.91–1.29)	0.346	
Apgar scores at 5 minutes	0.72 (0.44–1.17)	0.185	
Cord pH	0.01 (0.00–6.60)	0.165	
Lactate	1.36 (0.98–1.89)	0.062	
Base deficit	0.89 (0.69–1.14)	0.351	
Mode of delivery (emergency)	0.79 (0.03-23.89)	0.894	
Suspected foetal distress in labour (yes)	0.16 (0.00–5.54)	0.314	
Assisted ventilation at 10 minutes of age (yes)	12.38 (1.02–149.88)	0.048	

^a^ HRV variable was standardised prior to conducting the logistic regression.

^b^ HRV variable was log transformed (log base 10) and then standardised prior to conducting the logistic regression.

^c^ The odds ratio represents the change in odds for a 100g increase in birthweight.

HRV, Heart Rate Variability; EEG, electroencephalography; NN interval, normalised RR interval; HF power, high frequency power (0.15-0.4 Hz), LF/HF ratio, low frequency/high frequency ratio; MSE, multiscale entropy.

## Discussion

Using a large multicentre European dataset, the current study is the first to develop HRV and clinical models that accurately predict EEG grade in neonatal HIE. Early HRV analysis at median age of 5.9 h showed that high mean NN and low LF/HF rate, TINN, MSE complexity index, MSE short-scale slope and maximum MSE were associated with a worse encephalopathic EEG grade. When these HRV features were combined into a prediction model, the performance improved beyond individual features. The clinical model had a similar predictive value to the HRV model. However, the model with the best prediction for EEG background severity was the combined HRV and clinical model, with a AUROC 0.895 (95% CI:0.832–0.958). Although therapeutic hypothermia and anti-seizure medication have been demonstrated to impact EEG background and HRV, when the analysis was adjusted for these confounders, the model performance did not change (28–31).

It is well known that EEG background is an excellent predictor of brain injury and long-term outcomes in neonatal HIE (32–34). Neonatal EEG monitoring was also used as screening tool for initiation of therapeutic hypothermia in neonatal encephalopathy (35). Although EEG monitoring can be a relatively accessible neuromonitoring tool in developed countries, due to the high costs associated with specialised equipment and neurophysiological expertise, it is not as readily available in low-income countries (10, 12, 13, 36). On the other hand, ECG monitoring is less expensive, widely used and can be performed with good accuracy antepartum, during labour and very early in the delivery room. Therefore, HRV analysis might be very useful as an early tool to assess severity in neonatal HIE (37, 38).

Since the 1980′s, HRV has been investigated in different conditions as a non-invasive assessment tool for autonomic function (39, 40). Specifically for neonatal HIE, individual HRV features have been shown to be good predictors of encephalopathy severity, abnormal EEG background and outcome (14–16, 19). The current study has shown that a high mean NN (lower heart rate) was associated with moderate-severe EEG grade (OR 1.97 (95% CI:1.30–2.97), *p* = 0.001). However, when the analysis was adjusted for cooling status the difference did not reach statistical significance (data not presented). TINN was significantly lower in the moderate-severe group compared to the normal-mild EEG group (OR 0.66 (95% CI:0.45–0.97), *p* = 0.035), regardless of cooling status. A lower LF/HF ratio was associated with a more severe EEG grade (OR 0.40 (95% CI:0.25–0.64), *p* < 0.001). These findings, also confirmed previously by Goulding et al.0 (19, 28) in infants from a single centre, demonstrated a reduction in autonomic nervous system activity with an increase in HIE severity.

Furthermore, MSE complexity index, MSE short-scale slope and MSE maximum were significantly lower in the moderate-severe group compared with normal-mild EEG group, suggesting a decrease in HRV complexity with an increase in HIE severity, likely due to suppression in autonomic control.

Consistent with the literature (41, 42), this study showed that some clinical parameters were associated with worse EEG grade (low Apgar scores, need for assisted ventilation at birth), but the combination of clinical parameters increased the predictive value (clinical model AUROC 0.836 (95% CI:0.759–0.914)). The HRV prediction model performance was similar to the clinical model (AUROC 0.837 (95% CI:0.759–0.914)) in predicting severity of encephalopathy. However, the model combining early HRV features and readily available clinical information within 6 h of age had the best performance, with AUROC 0.895 (95% CI:0.832–0.958). Performance was similar when the analysis was performed using exclusively infants undergoing therapeutic hypothermia and infants without anti-seizure medication given prior to EEG epoch. The presented models could be very useful for settings where EEG is not readily available. These could have a greater impact especially in community hospitals and in low-income countries where neonatal EEG monitoring and expertise is scarce.

Several limitations must be considered. This was a secondary analysis of data collected for studies focused on neonatal seizures. To ensure only good quality recording was used for this analysis, all epochs were visually inspected and artefacts were removed before performing the HRV analysis. However, artefacts could be problematic if ECG recordings are used in real-time, at the bedside. Real-time monitoring of HRV would require the integration of an artefact detection algorithm. At the time of the selected epoch, some infants were already under hypothermia treatment (*n* = 97 infants) and some had already received anti-seizure treatment for clinically suspected seizures (*n* = 19 infants). To account for the confounding effect of hypothermia and anti-seizure medication, we also performed the analysis including only those infants that were cooled and which did not receive anti-seizure medication prior to selected epoch. These subgroup analyses showed no change in the model performance. However, we did not collect data on inotropic treatment. Due to missing data for pH, lactate and base deficit we did not include these parameters in the main analysis. However, we did develop the same models in infants which had this information (71 infants) and the prediction did not improve.

Despite these limitations, this study included early EEG and ECG data from a large neonatal population with all grades of HIE. Continuous EEG monitoring which is the gold standard recommended by current guidelines was used in this study for background EEG assessment (10). We are aware that most Neonatal Units do not have the resources to perform conventional EEG monitoring and are using aEEG monitoring for infants with HIE undertaking therapeutic hypothermia. Although this study analysed conventional EEG monitoring, current aEEG monitors can display two or three raw EEG channels from which the background analysis can be extracted.

We have shown that HRV and clinical data combined in a model using logistic regression analysis is an excellent predictor of EEG grade in the early newborn period and may be a very useful additional tool for neonatologists who are often faced with challenging decisions about therapeutic hypothermia, where EEG monitoring is not available or feasible.

In summary, our results are promising and suggest that an early combined HRV and clinical model could be useful to assess the severity of newborn encephalopathy in the early postnatal period. This might be helpful as a proxy marker for injury severity in settings where EEG monitoring is not available or not easily accessible; on the other hand, clinical information is readily available and ECG monitoring is non-invasive, easily available and unexpensive. Future work will require for this model to be validated in real time at the cot side and in all settings.

## Data Availability

The datasets presented in this article are not readily available because the clinical data is collected under a written proxy consent from the participants' guardians/parents, which did not include permission for sharing or open data. To be allowed to share this data under Irish Health Research Regulations we are required to re-consent families or to obtain approval by the Health Regulation Consent Declaration Committee. Requests to access the datasets should be directed to corresponding author.

## ANSeR consortium

INFANT Research Centre and Department of Paediatrics and Child Health, University College Cork, Cork, Ireland: Andreea M Pavel, Eugene Dempsey, Vicki Livingstone, Elena Pavlidis, Liudmila Kharoshankaya, Sean R Mathieson, Liam Marnane, Gordon Lightbody, Jackie O'Leary, Mairead Murray, Jean Conway, Denis Dwyer, Andrey Temko, Taragh Kiely, Anthony C Ryan, Geraldine B Boylan. Institute for Women's Health, University College London, London, UK: Janet M Rennie, Subhabrata Mitra. Utrecht Brain Center, University Medical Center Utrecht, Utrecht University, The Netherlands: Linda S de Vries, Lauren C Weeke, Mona C Toet. Department of Neonatal Medicine and Division of Paediatrics, Department CLINTEC, Karolinska University Hospital, Karolinska Institutet, Stockholm, Sweden: Mikael Finder, Mats Blennow, Ingela Edqvist. Rotunda Hospital, Dublin, Ireland: Adrienne Foran, Raga Mallika Pinnamaneni, Jessica Colby-Milley. Royal London Hospital and Queen Mary University of London, London, UK: Divyen K Shah, Nicola Openshaw-Lawrence. Department of Clinical Neurophysiology, Great Ormond Street Hospital for Children NHS Trust, London, UK: Ronit M Pressler. Homerton University Hospital NHS Foundation Trust, London, UK: Olga Kapellou. Clinical Neurophysiology, University Medical Center Utrecht, Utrecht, The Netherlands: Alexander C van Huffelen.
